# Circular and L50-like leaderless enterocins share a common ABC-transporter immunity gene

**DOI:** 10.1186/s12864-023-09750-2

**Published:** 2023-10-24

**Authors:** Claudia Teso-Pérez, Manuel Martínez-Bueno, Juan Manuel Peralta Sánchez, Eva Valdivia, María Esther Fárez-Vidal, Antonio Manuel Martín-Platero

**Affiliations:** 1 Department of Microbiology, University of Granada, Avda. Fuentenueva, s/n, Granada, 18071 Spain; 2 Department of Zoology, University of Seville, Avda. Reina Mercedes, 6, Seville, 41012 Spain; 3 Department of Biochemistry and Molecular Biology III and Immunology, School of Medicine, University of Granada, Granada, 18016 Spain; 4 Biomedical Research Institute of Granada, University Hospital Complex of Granada, University of Granada, Granada, 18071 Spain

**Keywords:** *Enterococcus*, ABC transporter, Cross-resistance, Enterocin MR10A/B, Enterocin AS-48, Carnocyclin

## Abstract

Microbes live within complex communities of interacting populations, either free-living in waters and soils or symbionts of animals and plants. Their interactions include the production of antimicrobial peptides (bacteriocins) to antagonize competitors, and these producers must carry their own immunity gene for self-protection. Whether other coexisting populations are sensitive or resistant to the bacteriocin producer will be key for the population dynamics within the microbial community. The immunity gene frequently consists of an ABC transporter to repel its own bacteriocin but rarely protects against a nonrelated bacteriocin. A case where this cross-resistance occurs mediated by a shared ABC transporter has been shown between enterocins MR10A/B and AS-48. The first is an L50-like leaderless enterocin, while AS-48 is a circular enterocin. In addition, L50-like enterocins such as MR10A/B have been found in *E. faecalis* and *E. faecium*, but AS-48 appears only in *E. faecalis*. Thus, using the ABC transporter of the enterocin MR10A/B gene cluster of *Enterococcus faecalis* MRR10-3 as a cross-resistance model, we aimed to unravel to what extent a particular ABC transporter can be shared across multiple bacteriocinogenic bacterial populations. To this end, we screened the MR10A/B-ABC transporters in available microbial genomes and analyzed their sequence homologies and distribution. Overall, our main findings are as follows: (i) the MR10A/B-ABC transporter is associated with multiple enterocin gene clusters; (ii) the different enterocins associated with this transporter have a saposin-like fold in common; (iii) the Mr10E component of the transporter is more conserved within its associated enterocin, while the Mr10FGH components are more conserved within the carrying species. This is the least known component of the transporter, but it has shown the greatest specificity to its corresponding enterocin. Bacteriocins are now being investigated as an alternative to antibiotics; hence, the wider or narrower distribution of the particular immunity gene should be taken into account for clinical applications to avoid the selection of resistant strains. Further research will be needed to investigate the mechanistic interactions between the Mr10E transporter component and the bacteriocin as well as the specific ecological and evolutionary mechanisms involved in the spread of the immunity transporter across multiple bacteriocins.

**Supplementary Information:**

The online version contains supplementary material available at 10.1186/s12864-023-09750-2.

## Background

Microbes usually live in complex communities composed of numerous interacting populations [1, 2], and thousands of microbial species can be found in the human gut [3] or in a single drop of water [4]. A complex network of interactions drives the community structure in this crowded scenario [5], including antagonism [6], which plays a major role in shaping the balance between populations. In the case of human and animal symbionts, the nature of the population established is crucial to its effect on the host. In this way, gram-positive *Enterococcus faecalis*, a habitual component of the human microbiota [7], can have a positive (commensal) or negative (pathogenic) effect on the host according to the enterococcal population. Some strains can improve the health of the host [8], but others are recognized as important opportunistic pathogens [9]. For instance, strains that produce cytolysin (an antimicrobial peptide) have been associated with greater hepatic damage in patients with alcoholic hepatitis [10].

The production of bacterial antimicrobial peptides (bacteriocins) is a common trait of enterococci (also referred to as enterocins) [11]. These antimicrobials generally act against close relatives, although some show a wide antimicrobial spectrum [12] and may therefore play an important role in driving niche colonization by specific populations and in establishing the final community structure. In this way, enterocin-producing *E. faecalis* populations have been found to outcompete other enterococcal populations in colonizing mammal gut microbiota. Kommineni and coworkers [13] reported that an *E. faecalis* producer of bacteriocin 21 (identical to AS-48 enterocin) was able to replace indigenous enterococcal populations in mice. This competitive exclusion implies that indigenous populations are susceptible to the bacteriocin produced. However, elucidation of the population dynamics and production/resistance patterns among wild populations requires knowledge of patterns of resistance to bacteriocins, which is currently very limited.

Bacteriocin producers must carry their own immunity gene to avoid self-inhibition [14]. It is usually grouped within the gene cluster for bacteriocin production, located on chromosome, plasmid, or other mobile genetic elements [15]. Various immunity mechanisms are involved in self-protection, such as specific membrane immunity proteins or proteases or multidrug transporters [16], including multicomponent ATP-binding cassette (ABC) transporters, which form part of the immunity/resistance system in several bacteriocins. This is the case for circular bacteriocins (such as the aforementioned AS-48), in which immunity plays a role in resistance, as well as the ABC transporter that expels the bacteriocin from the cell [17].

In addition to their producers, nonproducers can also display resistance to bacteriocins, which can be broadly classified as acquired or innate resistance. The latter is intrinsic to particular genera or species and is due to the presence of ‘orphan immunity genes’, bacteriocin degradation mechanisms, D-alanylation of teichoic acids of the cell wall, L-lysinylation of cell membrane phospholipids, or the growth phase in which the bacteria are found [15, 18, 19]. Acquired resistance can be generated by the spontaneous mutation of associated genes involved in the expression of specific receptors, cell wall synthesis, transcriptional regulation or energy metabolism and transport or by alteration in bacterial cell membrane hydrophobicity due to changes in its fatty acid composition or membrane receptors [15, 20]. Importantly, bacteria can not only be resistant but also develop coresistance and cross-resistance to one or two types of bacteriocins; however, there are few reports on this issue in the literature, and the results have been contradictory. Coresistance mainly appears when a producer strain is resistant to another bacteriocin of the same class, while cross-resistance takes place when a bacterial strain develops resistance to a different class of bacteriocin or to antibiotics [20]. However, the latter has largely been observed by experimental manipulation [21], and little is known about cross-resistance in wild populations.

Our group previously reported cross-resistance between *E. faecalis* MRR10-3 and *E. faecalis* A-48-32 [22], which produce bacteriocins of distinct classes, i.e., enterocins MR10A/B (class II- not posttranslationally modified) and enterocin AS-48 (class I- circular posttranslationally modified), respectively. Both strains share a homologous ABC-type transport system (Mr10EFGH and As-48EFGH, respectively) involved in self-immunity and cross-resistance [22]. AS-48 has an additional ABC transporter downstream the As-48EFGH transporter [23], and a second transporter involving pleckstrin homology domains was recently described in L50-like enterocins downstream the Mr10EFGH transporter [24], but only the As-48EFGH and Mr10EFGH transporters show similarity between the transporters present on both enterocin gene clusters [22]. ABC transporters are widespread in multiple organisms and generally comprise four core domains: two transmembrane domains (TMDs) and two hydrophilic and peripheral cores, where Walker A and Walker B motifs and the C-loop and the D-loop (two hallmarks of transporters) are associated with the cytoplasmic surface of the membrane [25]. Periplasmic binding proteins are needed in some cases to confer affinity, specificity, and directionality to the ABC transporter, as well as peripheral or auxiliary proteins [26]. In the case of the ABC transporter shared by both gene clusters, recent studies on EntDD14, a L50-like leaderless enterocin, have described a dual role for this transporter, which is involved in both self-immunity and export of the enterocin [27]. However, knowledge on the role of each individual component remans limited. The functions of Mr10F, Mr10G, and Mr10H components (accessory adaptor, ATP binding domain and permease, respectively) have well-documented functions in transport and energy supply, but the role of the Mr10E component (a transmembrane protein) is still unknown. It was reported to act in the assembly of the other three components in a structurally similar ABC transporter in *Bacillus subtilis*, while a role in protection against sporulation-delaying protein (SDP) toxin was also proposed [28].

The aim of this study was to determine whether the ABC transporter involved in MRR10-3 and A-48-32 cross-resistance was widespread among wild populations. A search of the ABC transporter in public databases revealed its presence in multiple enterococcal populations associated with different bacteriocins and enterococcal species and a low frequency of orphan strains.

## Results

### ABC transporter taxonomic distribution

The Mr10EFGH ABC transporter involved in MR10A/B self-immunity proved to be specific to the genus *Enterococcus*, especially to *E. faecalis* and *Enterococcus faecium*. Homologous genes for each of the four transporter components appear in a wide range of genera, although most of this range is attributable to the wide distribution of Mr10G and Mr10H, i.e., the ATP binding domain and permease, respectively. Nevertheless, a fine-grained screening to maximize true positives (with > 40% identity and > 70% coverage) limited homologous proteins to this transporter to 14 genera (Table 1). The taxonomic range of Mr10E is narrower, limited to the genus *Enterococcus* alone; therefore, only *Enterococcus* showed complete ABC transporters homologous to Mr10EFGH (Table 1) when each transporter component was checked against its expected position. The similarity of each transporter component found among *Enterococci* varied depending on the gene. Thus, *mr10E* showed a similarity to all strains close to 45%, while *mr10F*, *mr10G*, and *mr10H* showed similarities of approximately 75–99%.

**Table 1:** Genera with proteins homologous to each component of the Mr10EFGH transporter

Genus	Mr10E	Mr10F	Mr10G	Mr10H	Complete ABC transporter
*Alkalibacterium*	0	0	0	1	0
*Carnobacterium*	0	2	8	3	0
*Clostridioides*	0	0	8	2	0
*Desemzia*	0	0	0	1	0
*Floricoccus*	0	2	2	2	0
*Garciella*	0	0	0	2	0
*Granulicatella*	0	0	0	1	0
*Lactobacillus*	0	0	0	12	0
*Lactococcus*	0	1	1	10	0
*Marinilactibacillus*	0	0	0	6	0
*Sedimentibacter*	0	0	2	1	0
*Streptococcus*	0	1	2	1	0
*Trichococcus*	0	0	20	13	0
*Enterococcus*	85	130	756	390	85

The full transporter was solely found in *E. faecalis* (x29), *E. faecium* (x47), *E. durans* (x2), *E. phoeniculicola* (x3), *E. hirae* (x1), and *Enterococcus sp*. (x3).

### ABC transporter genomic distribution

**Fig. 1:**
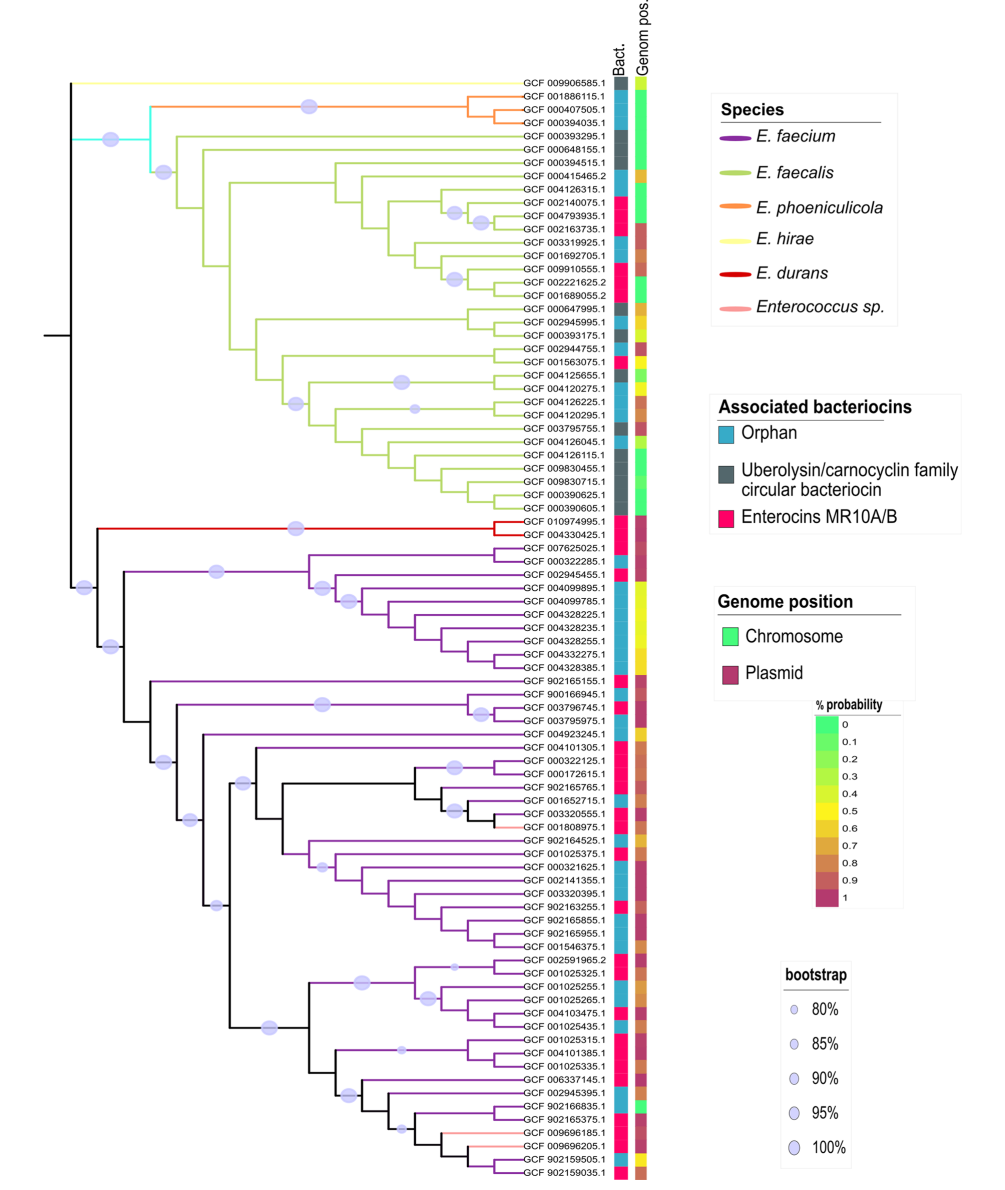
Mr10EFGH transporter phylogenetic tree. The image represents the phylogenetic tree of the ABC transporter of the mr10A/B gene cluster. From left to right, the first annotation bar represents the bacteriocin associated with the MR10A/B transporter. Three cases can be observed: the orphan MR10A/B transporter in bacteriocin (light blue), associated with circular bacteriocins (gray) or associated with leaderless bacteriocins (pink). The second bar represents the plasmid (violet) or chromosome (green) position of the contig in the genome. The color of the branches represents the species associated with each case. The majority of species with this transporter are *E. faecalis* (light-green) and *E. faecium* (violet), although it also appears in *E. phoeniculicola* (orange), *E. hirae* (light-yellow), *E. durans* (red), and *Enterococcus sp.* (light pink). The gray dot located on the tree’s branches represents the bootstrap value between 80 and100%)

**Fig. 2:**
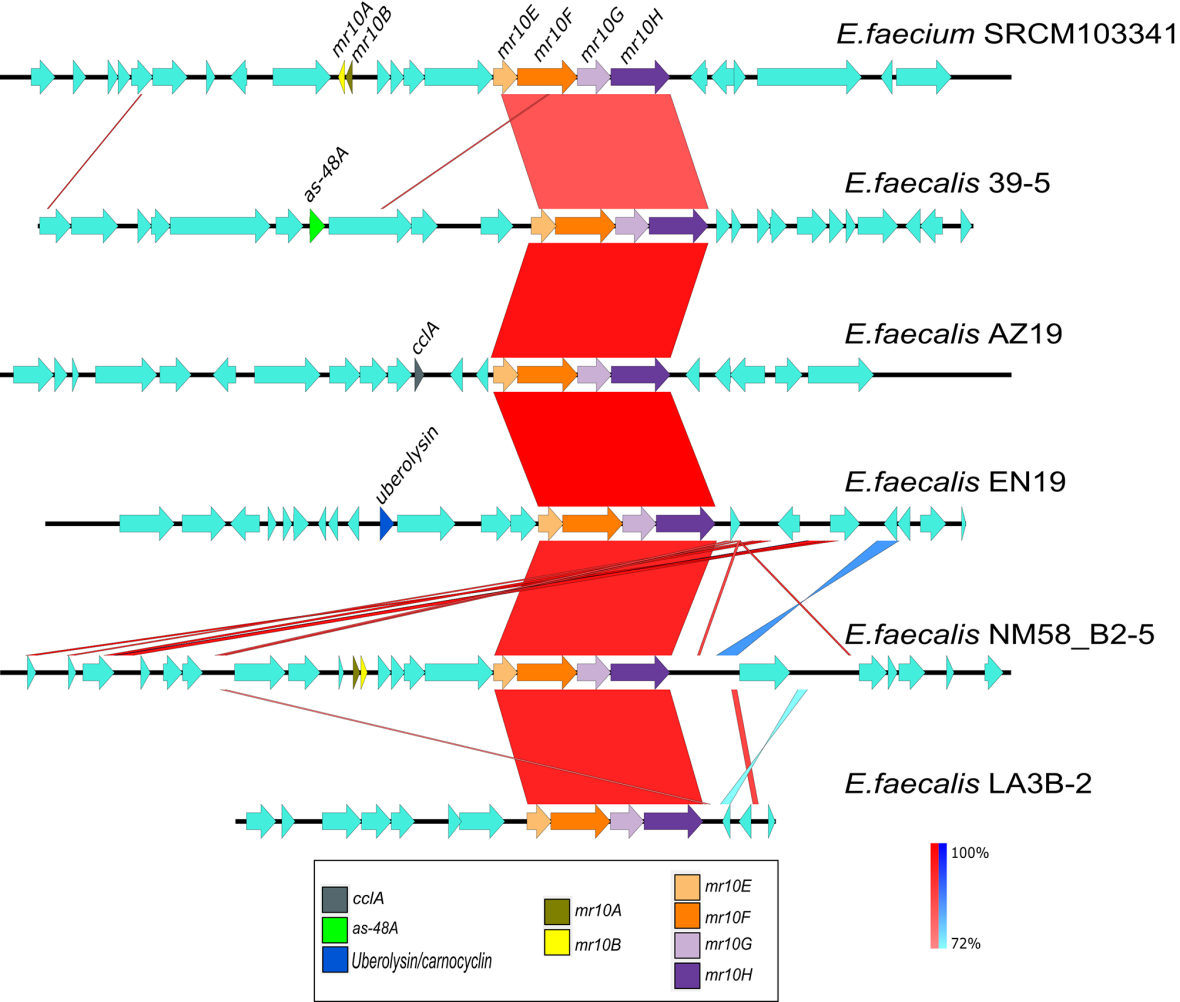
Comparison between different bacteriocin gene clusters. The figure shows the homologies found when comparing contigs from different species that contain the Mr10EFGH transporter associated with the structural genes of enterocins MR10A/B (yellow and gold arrows), AS-48 (green arrow), carnocyclin A (gray arrow), uberolysin enterocin family (blue arrow) and orphans in bacteriocins. Similarities are conserved only at the ABC transporter level. From top to bottom, GenBank accession nos. NZ_CP035137.1 (*Enterococcus faecium* SRCM103341), NZ_KB944733.1 (*Enterococcus faecalis* EnGen0369 39 − 5), NZ_AYLU01000046.1 (*Enterococcus faecalis* AZ19), NZ_PJXM01000014.1 (*Enterococcus faecalis* EN19), NZ_SRYT01000012.1 (*Enterococcus faecalis* NM58_B2-5) and NZ_KE352861.1 (*Enterococcus faecalis* LA3B-2)

The *mr10EFGH* ABC transporter involved in MR10A/B self-immunity was found to be widespread among *E. faecalis* and *E. faecium* (Table 1; Fig. 1), although it showed specific variations according to the carrier species and genetic background. The reference transporter was from *E. faecalis* within the *mr10A/B* gene cluster for the production of enterocins MR10A/B, but it was associated with three other types of bacteriocins (i.e., AS-48, uberolysin/circularin family circular bacteriocin, and carnocyclin A) and even with orphans of any bacteriocin structural gene (Fig. 2).

**Fig. 3:**
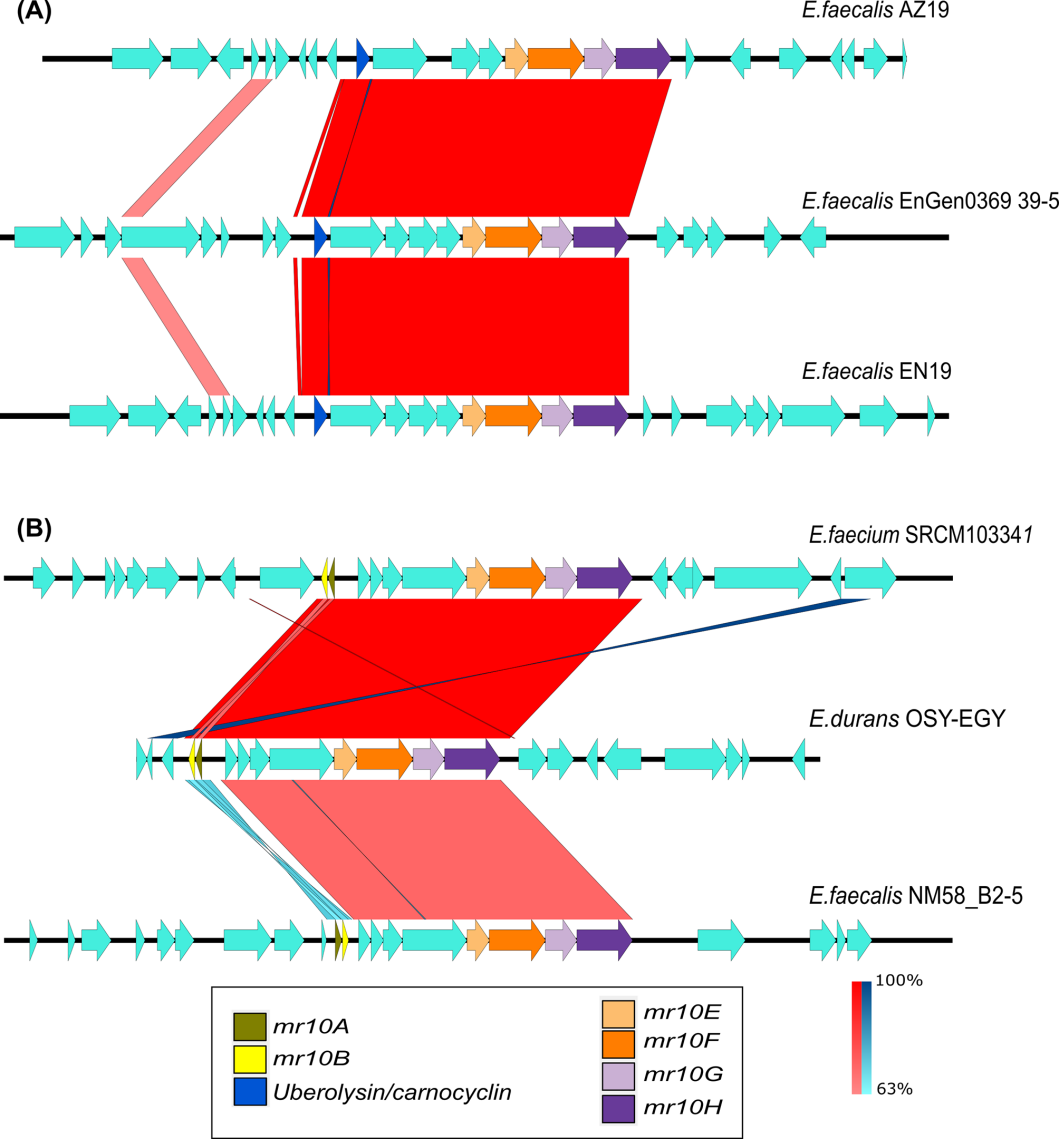
Comparison between contigs containing the transporter Mr10EFGH associated with the same bacteriocin. Panel A shows the comparison between contigs that contain the ABC transporter associated with structural genes of the uberolysin enterocin family (blue arrow). Panel B shows the similarity between contigs that contain the ABC transporter associated with structural genes of MR10A/B enterocins (yellow and gold arrows). In both cases, the homologies are maintained at the gene cluster level. The similarity varies between 63% (light red) and 100% (dark red). From top to bottom, GenBank accession nos. are NZ_AYLU01000046.1 (*Enterococcus faecalis* AZ19), NZ_KB944733.1 (*Enterococcus faecalis* EnGen0369 39 − 5), NZ_PJXM01000014.1 (*Enterococcus faecalis* EN19), NZ_CP035137.1 (*Enterococcus faecium* SRCM103341), NZ_SEHG01000089.1 (*Enterococcus durans* OSY-EGY), and NZ_SRYT01000012.1 (*Enterococcus faecalis* NM58_B2-5)

These other bacteriocins are class I circular bacteriocins, whereas MR10A/B and MR10-like bacteriocins (such as L50A/B) are class II leaderless bacteriocins. Within each bacteriocin type, its corresponding gene cluster was conserved, but only the Mr10EFGH transporter was shared between them (Figs. 2 and 3). Therefore, this transporter did not show strong conservation of its genetic background either up- or downstream. Only gene clusters for bacteriocin production of the same type were conserved (Fig. 3).

However, the level of variation was considerably higher in *E. faecalis* than in *E. faecium*. While the transporter was associated with four different bacteriocins in *E. faecalis*, it was solely associated with MR10-like bacteriocins in *E. faecium* (Fig. 2). In addition, there were a few cases of orphan transporters in both species. In some cases, these orphan genomes were the result of incomplete contigs, e.g., NZ_LWHF01000045.1 (*Enterococcus faecium* strain 17OM39), NZ_PKMN01000041.1 (*Enterococcus faecalis* strain EN788), or NZ_LDND01000117.1 (*Enterococcus faecium* strain KACC15711); however, they were evident orphan transporters in some cases, i.e., GenBank accession no. NZ_CABHDR010000049.1 (*Enterococcus faecium* strain 4928STDY7387731), GenBank accession no. NZ_KE352861.1 (*Enterococcus faecalis* LA3B-2 Scaffold63), or GenBank accession no. NZ_KB946329.1 (*Enterococcus phoeniculicola* ATCC BAA-412 acvKl-supercont1.7). In addition, the transporter is placed on both chromosomes and plasmids in these cases, whereas it is only observed on plasmids in *E. faecium*, and there is no specific pattern of placement in *E. faecalis* (Fig. 1).

### Molecular evolution of the Mr10EFGH ABC transporter

**Fig. 4:**
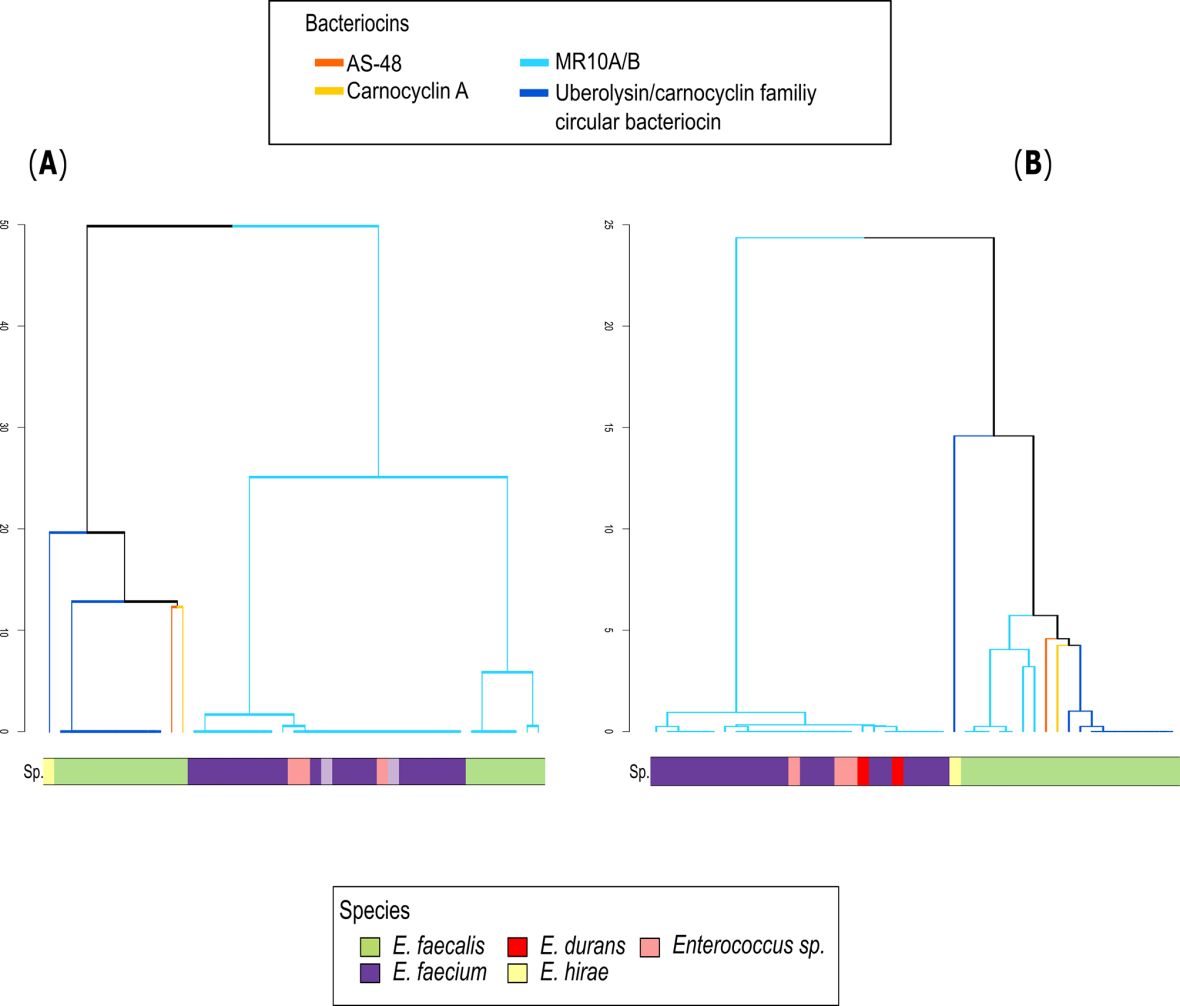
Protein similarity between the different carriers of Mr10E (**A**) and Mr10F (**B**) proteins. The color of the branches represents the bacteriocin associated with each ABC transporter: light blue = enterocins MR10A/B, dark blue = uberolysin, yellow = carnocyclin A, and orange = AS-48. The color bar represents the strain corresponding to each contig: light-green = *E. faecalis*, violet = *E. faecium*, red = *E. durans*, yellow = *E. hirae*, and pink = *Enterococcus sp*. Mr10E shows greater similarities with those associated with the same bacteriocin, while Mr10F shows higher similarities with those from the same species

In addition to the differential distribution of the *mr10EFGH* ABC transporter, each transporter component showed different amino acid sequences according to the bacteriocin structural gene or the bacterial species. Specifically, the primary sequence of Mr10E was constrained by its associated bacteriocin, while Mr10F, Mr10G, and Mr10H were more conserved within each enterococcal species (Fig. 4). In other words, Mr10E was more similar between those in the same bacteriocin, while Mr10F, Mr10G, and Mr10H were more similar between the same enterococcal species, independent of the associated enterocin. Hence, Mr10F, Mr10G, and Mr10H phylogenies agree with the enterococcal taxonomy, while Mr10E agrees with the phylogeny of the enterocin structural gene (Fig. 4).

**Fig. 5:**
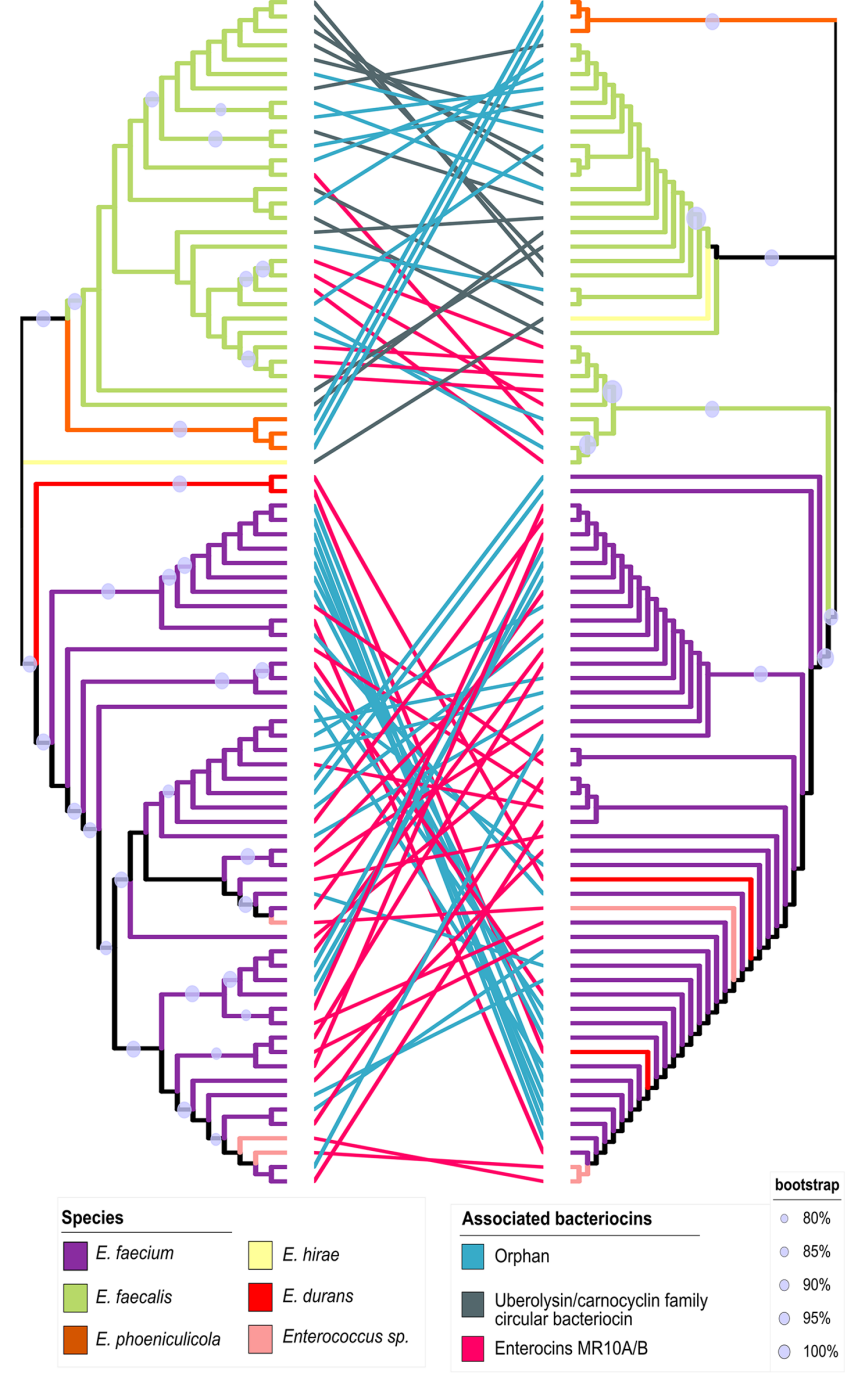
Tanglegram between genomes carrying the Mr10EFGH transporter and their *mr10E* gene. Maximum-likelihood phylogenetic tree of the core genomes (left) and *mr10E* gene (right). The color of the branches represents the species: violet = *E. faecium*, light-green = *E. faecalis*, orange = *E. phoeniculicola*, light-yellow = *E. hirae*, red = *E. durans*, and light-pink = *Enterococcus sp.* The color of the connecting lines represents the bacteriocin associated with the Mr10EFGH transporter: pink = enterocins MR10A/B, gray = circular bacteriocins, and blue = orphan transporter in bacteriocins

**Fig. 6:**
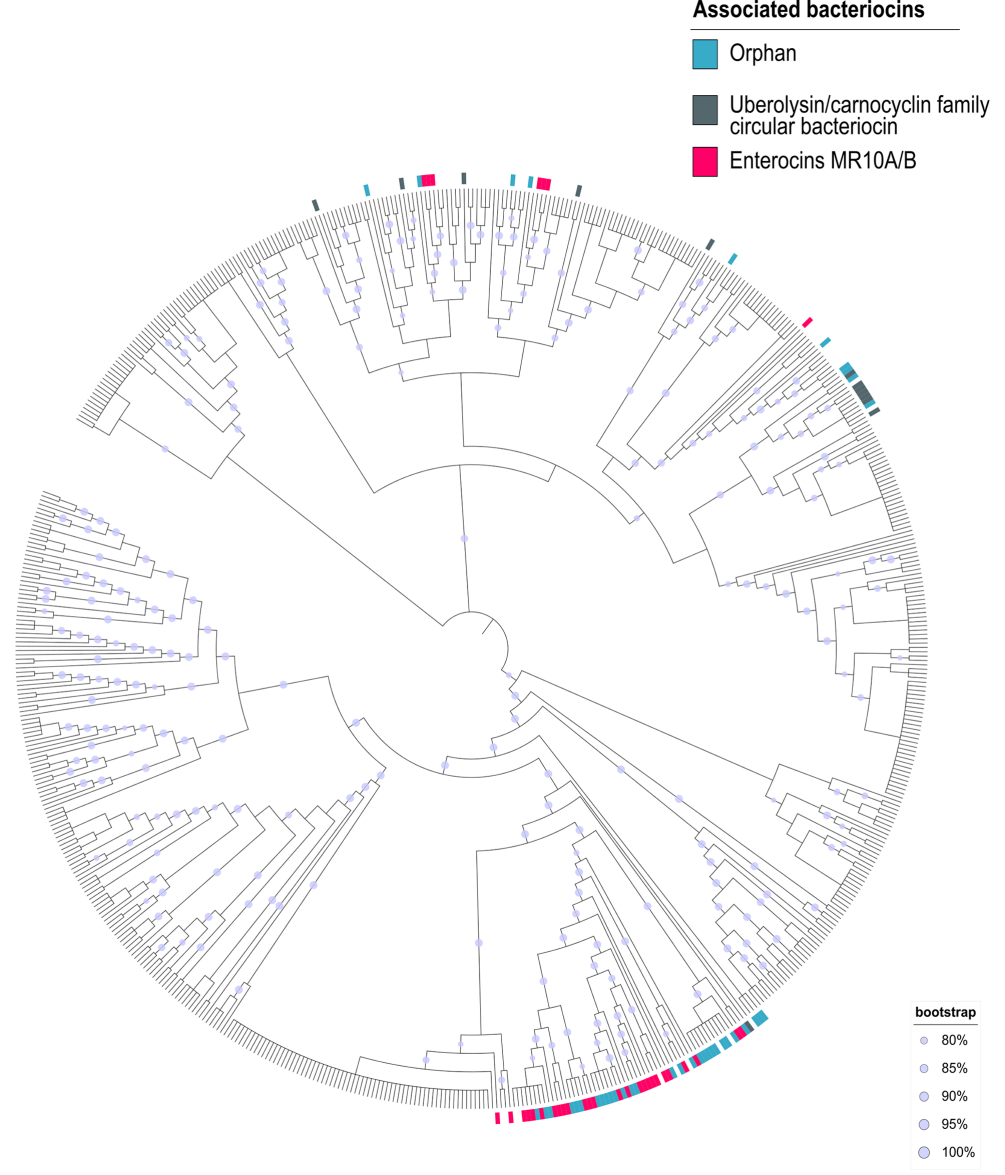
Maximum-likelihood phylogenetic tree of the core genomes from strains carrying the Mr10EFGH transporter. The outer bar represents the bacteriocin associated with the Mr10EFGH transporter; pink = enterocin MR10A/B enterocin, gray = circular bacteriocin, and blue = orphan ABC transporter. The gray dot located on the tree’s branches represents the bootstrap value (between 80 and100%)

A differential evolutionary history from the enterococcal population is observed for each component of the ABC transporter and for the complete enterocin gene cluster, as shown by the discrepancies between genome and transporter phylogenies (Fig. 5). Indeed, although there are some clades enriched in some enterocins, neither the enterocins nor the transporter form monophyletic clades (Fig. 6). This implies that there is no common ancestor that includes all of the same bacteriocins.

## Discussion

This study of the distribution of the *mr10EFGH* ABC transporter reveals some degree of modularity in the immunity genes associated with bacteriocin production. This transporter, involved in MR10A/B immunity/resistance, was found to be constrained to the *Enterococcus* genus but widespread among different enterocins and enterococcal species. Accordingly, bacteriocinogenic strains with this transporter could share a common immune system and generate a cross-resistance group of enterococcal populations, as previously shown for *E. faecalis* MRR10-3 and *E. faecalis* S-48. However, this group could be characterized by varying degrees of resistance among populations as a function of the enterocin structural gene and transporter specificity.

ABC transporters play multiple biological roles in numerous organisms and are classified into three main groups according to their involvement in the import of nutrients through the cellular membrane, mRNA translation and DNA repair, and the export or secretion of various molecules, including toxins [29]. This last function explains the frequent presence of ABC transporters as immunity genes in several bacteriocins. This is the case for the Mr10EFGH ABC transporter associated with the enterocins MR10A/B, which is also found in AS-48 producers and is involved in their cross-resistance [22]. According to the present findings, this transporter is also shared by other bacteriocins of the uberolysin/carnocyclin family and by some orphan strains of the enterocin structural gene (Fig. 1). Hence, sharing the immunity transporter among different bacteriocins may produce cross-resistance groups that could recognize and resist other groups to some degree. This cross-resistant group of enterococcal populations might share some structural characteristics that facilitate recognition of the shared ABC transporter, including a saposin-like protein domain [30]; a hydrophobic core of 4–5 α-helices with a predominantly hydrophobic outer surface; and solvent-exposed tryptophan or tyrosine residues close to the N- or C-termini [30]. In their study of the structure of enterocins 7 A and 7B, homologous to MR10A and MR10B, Lohans et al. [31] highlighted the resemblance of these enterocins to carnocyclin A (within the uberolysin/carnocyclin family) [31]. The question therefore arises whether other bacteriocins that share specific domains could also share a common immunity gene to form other potentially cross-resistance groups.

The potential cross-resistance group of enterococcal populations includes *E. faecalis*, *E. hirae*, *E. faecium*, *E. durans*, *Enterococcus sp.*, and *E. phoeniculicola*. Hence, in addition to its mobilization between different enterocin gene clusters, this transporter must have been mobilized between different species. Bacteriocin gene clusters are frequently plasmidic and therefore inherently mobile [14], but they have also been mobilized at chromosomes. For instance, the gene cluster of enterocin MR10 is chromosomal, whereas the homologous gene cluster of enterocin L50 is plasmidic [22, 32]. Furthermore, MR10 is produced by *E. faecalis*, but L50 is produced by *E. faecium* [22, 32]. The fact that the transporter in *E. faecium* appears solely in plasmids, is associated with only one bacteriocin, and has more phylogenetically coherent carrying strains in comparison to *E. faecalis* (Fig. 6) indicates that it may have acquired the transporter more recently. However, it should be noted that not only whole gene clusters but also the immunity gene alone can be mobilized, as evidenced by its presence in distinct bacteriocins and in some orphan strains. Recombination is a frequent mechanism for shaping enterococcal genome composition [33], and genetic exchange between strains of enterococci is responsible for their wide genomic diversity [34]. This genetic transfer is attributable to the presence of phages, plasmids, pathogenicity islands (PAI), and conjugative elements in the genome of enterococci [34]. In fact, up to 25% and 38% of the genomes of *E. faecalis and E. faecium*, respectively, are acquired elements [35, 36]. This set of mobile genetic elements, known as the mobilome, contributes to the plasticity of the genome and to the dissemination of antibiotic resistance and pathogenicity genes [37]. This last effect is especially important in relation to *E. faecium*, because antimicrobial resistance genes acquired by horizontal gene transfer are the main drivers of their selection and dissemination in the hospital setting [38]. Hence, resistance to these antimicrobial peptides in enterococcal populations can spread beyond enterocin producers, and some orphan transporters have also been identified, with important implications for the modeling of resistance dynamics in wild populations. The rock-paper-scissors model is frequently used to explain the dynamics between bacteriocin producers and resistant and susceptible populations [14]; however, this model has mainly been established by the mutagenesis of different populations [39]. In addition to mutagenesis, the present study shows that resistance can be transmitted without bacteriocin production in wild populations. Furthermore, theoretical models in a more realistic scenario with multiple interacting strains take into account the formation of units of mutually immune species [40]. Our results could shed light on the molecular basis of such units by these potential cross-resistance groups by sharing a common immunity gene. The scenario is more complex, however, because differences in specificity between structural and immunity genes (see below) could generate populations with distinct degrees of resistance. Future competition experiments with these differential populations are needed to elucidate the dynamics of producers and resistant and susceptible populations within wild microbial communities.

In addition to the variation in the transporter among enterococcal populations, each ABC transporter component shows a different specificity. The components Mr10G and Mr10H correspond to the ATP binding domain and permease, respectively, which are the most conserved components of the different ABC transporters [25]. This explains why Mr10G and Mr10H were found in several species, whereas Mr10E and Mr10F were observed only in *Enterococcus* (Table 1). Mr10E and Mr10F components correspond, respectively, to a transmembrane protein and an accessory adaptor. The function of Mr10E remains unknown, but it is known to contain four transmembrane α-helices [23]. Mr10F displays similarities with the efflux resistance-nodulation-division transporter periplasmic adaptor subunit [22]. It is typical of gram-negative bacteria, facilitating the transport of various substrates through the outer membrane, but it has also been found in gram-positive bacteria, although its function is not clear. Mr10E and Mr10F were therefore found in a much narrower range of bacteria, i.e., specific enterococcal populations (Table 1), although Mr10F, Mr10G, and Mr10H were well conserved within each enterococcal species, suggesting a more specific interaction with other cell features. In contrast, Mr10E was more conserved within the same enterocin gene cluster, regardless of the producer species (Figs. 4 and 5). Although transporters equivalent to that of Mr10EFGH have been found in other bacteriocin clusters, the function of the Mr10E protein has not been established, as noted above. The present results indicate a direct interaction between the bacteriocin and the Mr10E component of the transporter, ultimately producing the specificity of the resistance. This specific interaction might play an important role in the previously observed differential susceptibility pattern between MR10 and AS-48, given the difference in Mr10E between them (i.e., a 46% identity).

## Conclusions

A balanced microbial composition is key for microbial community functioning. For instance, multiple diseases are associated with dysbiosis of the host microbiota. This composition is fine-tuned by microbial interactions, with antagonistic interactions playing a major role. An understanding of the rules governing microbial assembly and dynamics is therefore crucial for constructing precise models of microbial dynamics and evolution. In the present study, we have mainly found that the immunity MR10A/B-ABC transporter is associated with multiple enterocin gene clusters. There is a wide variety of bacteriocins among bacteria, but we only found this immunity transporter in circular and L50-like leaderless bacteriocins carried by *Enterococcus*, being observed in multiple populations of *Enterococcus faecalis* and *Enterococcus faecium*. The L50-like leaderless enterocins are substantially conserved, while the circular enterocins show a greater variation, but all enterocins sharing a homologous immunity gene have a saposin-like fold in common. In addition, the Mr10E component of the transporter, whose function is the least well known, proved to be the most specific component for these enterocins, pointing to a key role in transporter-enterocin interactions. Taking into account the presence of a common resistance system among different bacteriocingenic populations will be crucial to develop more precise predictive models of production/resistance dynamics in the wild. Further research will have to investigate to what extent these findings apply to other bacteriocin immunity systems and the specific evolutionary mechanisms involved in the transference of the immunity transporter.

## Methods

### Mr10EFGH ABC transporter screening

To analyze the distribution of the enterocin immunity Mr10EFGH ABC transporter, a search for its homologs was undertaken in public databases, first exploring taxa that might carry an ABC transporter homologous to Mr10EFGH. A search was also conducted for protein sequences encoded by *mr10E, mr10F, mr10G*, and *mr10H* belonging to the MR10 gene cluster (GenBank accession no. MW689545) at the National Center for Biotechnology Information (NCBI) using blastp (version 2.10.0+; max target sequences: 1000; expect threshold: 1e^− 6^) [41] on the non-redundant protein sequence database (as available on 06/04/2020) among *Enterococcus* and non-*Enterococcus* species. Next, the full genetic background of ABC transporters was obtained by downloading all genomes from NCBI RefSeq (NCBI Reference Sequence Database) (06/04/2020) corresponding to the genera that showed a hit with any of the *mr10EFGH* genes. This procedure yielded 64,116 genomes corresponding to 121 genera (Supplementary Table S1), and a blast database of these genomes was created with makeblastdb (blast 2.10.1+) [42] and used for further analysis. A search was then made for ABC transporters homologous to Mr10EFGH in our own genomic database by running a tblastn for each protein (2.10.1+; Expect threshold: 1e^− 6^) [41]. False positives were minimized by considering only hits with more than 40% identity and 70% coverage as positive [43], defining hits placed at the expected position and distance (+/-500 bp) in each genome as full ABC transporters, using the *mr10* gene cluster as a reference [22]. It was also determined whether the transporter was located at a plasmid or chromosome by using PlasClass software to analyze its corresponding molecule/contig [44]. PlasClass uses four logistic regression models to classify sequences of different lengths and assigns class probabilities to each sequence. A sequence is classified as having a plasmid origin if the probability that it belongs to the plasmid class is > 0.5 [44].

### Analysis of Mr10EFGH ABC transporter association with bacteriocin structural genes

A search was conducted of bacteriocin structural genes in the genomic database of MR10EFGH-carrying genomes that we created (see above) to determine whether the *mr10EFGH* ABC transporter belonged to a genetic cluster involved in bacteriocin production. For this purpose, tblastn (version 2.10.1+) was run [45, 46] with a 10^− 6^ e-value threshold to search for the bacteriocins of the BACTIBASE database [47] plus a bacteriocin showing hits in a previous analysis (GenBank accession no. WP_002368637.1) with our previously generated genomic database. False positives were again minimized by defining only hits with more than 40% identity and 70% coverage as positive [43]. When several hits were obtained at the same position, the hit with the highest percentage identity was considered. Finally, the *mr10EFGH* ABC transporter was considered part of a bacteriocinogenic gene cluster when the bacteriocin structural gene was at the expected distance from the transporter (+/- 500 bp), using MR10A/B (GenBank accession no. MW689545), AS-48 (GenBank accession no. Y12234 and AJ438950), and the Carnocyclin A (GenBank accession no. NZ_AYLU01000046.1) bacteriocins gene clusters as references.

### Comparative genomic analysis of the Mr10EFGH ABC transporter genetic background

The level of synteny up- and downstream from the transporter was studied by comparing a region of 10,000 bp containing the *mr10EFGH* transporter using easyfig software (version 2.1) [48] between different groups of transporter-carrying strains.

### Phylogenetic analysis

The relationship between transporters observed in different genomic scenarios was studied by phylogenetic analysis of the transporter and corresponding populations. To this end, a phylogenetic tree was constructed with each gene independently (i.e., *mr10E, mr10F, mr10G*, and *mr10H*) and with the full transporter using the concatenated alignment of each individual gene. Each multiple sequence was aligned by applying the clustalW algorithm in MEGA-X (version 10.1.8) [49]. In all cases, a maximum likelihood tree was constructed with MEGA-X (version 10.1.8; N° Bootstrap Replications: 1000; Tamura-Nei model).

The mobility of the transporter was examined by constructing a phylogenetic tree of the enterococcal populations carrying the transporter. First, all genomes that hit with each transporter protein independently were annotated using Prokka (version 1.12) [50]. Next, the pangenome was calculated using Roary software (version 3.11.2) [51], which generates a multi-FASTA alignment of all core genes that are then used to construct the maximum likelihood phylogenetic trees with MEGA-X software (N° Bootstrap Replications: 1000; Tamura-Nei model) of all genomes with some component of the transporter and of those with the complete Transporter-2.

### Protein specificity

The protein specificity of each Mr10EFGH transporter protein was determined by cluster analysis. An identity matrix was first generated by multiple sequence alignment using Clustal Omega [52] and then converted into a distance matrix using R software (version 4.1.2) embedded in Rstudio software [53]. A dendrogram was constructed using the hclust function (“average” method) from the stats package [53]. RColorBrewer [54] and dendextend [55] packages were also used.

### Electronic supplementary material

Below is the link to the electronic supplementary material.

Supplementary Material 1

Supplementary Material 2

## Data Availability

The dataset used in this study is publicly available in Genbank under the accession numbers list in Supplementary Table S2. Additional files included in this article are also available in Supplementary Table S1.
