# Supplementary material for: Circular and L50-like leaderless enterocins share a common ABC-transporter immunity gene
Source: BMC Genomics. 2023 Oct 24;24:639. doi: 10.1186/s12864-023-09750-2 (PMC10598978; doi:10.1186/s12864-023-09750-2)
Supplement: Supplementary file 1 — Supplementary Material 1 [file 12864_2023_9750_MOESM1_ESM.docx]

**Supplementary Data**

**Table S1**. Bacterial genomes downloaded from NCBI. The table represents the bacterial genera that hit any of the four proteins of the Mr10EFGH transporter.

| Genus | Genomes |
| --- | --- |
| *Acetobacterium* | 14 |
| *Acidaminobacter* | 1 |
| *Acidiferrobacter* | 5 |
| *Acidobacterium* | 59 |
| *Aeribacillus* | 7 |
| *Aerococcus* | 69 |
| *Alkalibacterium* | 12 |
| *Alkaliphilus* | 6 |
| *Allofustis* | 1 |
| *Alloiococcus* | 2 |
| *Aminiphilus* | 1 |
| *Anabaena* | 9 |
| *Anaerofustis* | 4 |
| *Anaerolinea* | 2 |
| *Anaerosalibacter* | 4 |
| *Asaccharospora* | 1 |
| *Atopobacter* | 2 |
| *Bacillus* | 4808 |
| *Bacterium_CH2-D8-79* | 1 |
| *Bacteroides* | 1112 |
| *Bifidobacterium* | 1036 |
| *Caldicellulosiruptor* | 36 |
| *Caloranaerobacter* | 5 |
| *Calothix* | 16 |
| *Campylobacter* | 3302 |
| *Carboxydothermus* | 4 |
| *Carnobacterium* | 94 |
| *Catonella* | 1 |
| *Cetobacterium* | 5 |
| *Chlamydia* | 370 |
| *Chloroflexus* | 6 |
| *Cylindrospermum* | 4 |
| *Clostridioides* | 1864 |
| *Corallococcus* | 32 |
| *Corynebacterium* | 968 |
| *Curvibacter* | 5 |
| *Dendrosporobacter* | 1 |
| Genus | **Genomes** |
| *Desemzia* | 1 |
| *Desnuesiella* | 1 |
| *Desulfofarcimen* | 6 |
| *Desulfofundulus* | 6 |
| *Dolosicoccus* | 3 |
| *Dolosigranulum* | 14 |
| *Dorea* | 69 |
| *Enterococcus* | 3997 |
| *Eremococcus* | 2 |
| *Erwinia* | 194 |
| *Eubacterium* | 79 |
| *Finegoldia* | 27 |
| *Fischerella* | 41 |
| *Flacklamia* | 13 |
| *Floricoccus* | 2 |
| *Fusobacterium* | 183 |
| *Garciella* | 4 |
| *Gardnerella* | 102 |
| *Geobacter* | 26 |
| *Geomicrobium* | 4 |
| *Globicatella* | 4 |
| *Granulicatella* | 10 |
| *Hydrogenobacter* | 3 |
| *Ignavigranum* | 1 |
| *Lachnoclostridium* | 20 |
| *Lachnospira* | 189 |
| *Lacrimispora* | 12 |
| *Lactobacillus* | 2794 |
| *Lactococcus* | 283 |
| *Lagierella* | 2 |
| *Leuconostoc* | 184 |
| *Leptospirillum* | 9 |
| *Listeria* | 3321 |
| *Mahella* | 1 |
| *Marinilactibacillus* | 7 |
| *Marininema* | 2 |
| *Melghirimyces* | 3 |
| *Melisococcus* | 19 |
| *Methylopumilus* | 41 |
| *Miniphocibacter* | 1 |
| *Moorella* | 26 |
| *Mycobacteroides* | 1773 |
| *Nitrosomonas* | 56 |
| *Nitrospira* | 30 |
| Genus | **Genomes** |
| *Nostoc* | 49 |
| *Oenococcus* | 231 |
| *Paenibacillus* | 572 |
| *Paeniclostridium* | 51 |
| *Paraburkholderia* | 163 |
| *Paraclostridium* | 9 |
| *Peptostreptococcus* | 14 |
| *Phenylobacterium* | 13 |
| *Pilibacter* | 1 |
| *Pyxidicoccus* | 2 |
| *Rhodoferax* | 16 |
| *Romboutsia* | 11 |
| *Ruminococcus* | 199 |
| *Sedimentibacter* | 3 |
| *Smithella* | 18 |
| *Sphingobacterium* | 83 |
| *Sporolactobacillus* | 17 |
| *Staphylococcus* | 14053 |
| *Streptococcus* | 14796 |
| *Streptomyces* | 1737 |
| *Suicoccus* | 1 |
| *Synechococcus* | 81 |
| *SyntrophusSyntrophaceae* | 24 |
| *Tepidanaerobacter* | 3 |
| *Terrisporobacter* | 5 |
| *Thermacetogenium* | 2 |
| *Thermincola* | 2 |
| *Thermoanaerobacter* | 34 |
| *Thermoanaerobacterium* | 12 |
| *Thermodesulfovibrio* | 6 |
| *Thermosediminibacter* | 29 |
| *Trichococcus* | 18 |
| *Tissierellia* | 180 |
| *Tolypothrix* | 6 |
| *Vaginisenegalia* | 1 |
| *Vagococcus* | 32 |
| *Vibrio* | 4029 |
| *Virgibacillus* | 46 |
| *Weissella* | 132 |
| *Candidate division Zixibacteria* | 1 |
|  |  |
